# [Retracted] miR‑660‑5p is associated with cell migration, invasion, proliferation and apoptosis in renal cell carcinoma

**DOI:** 10.3892/mmr.2024.13210

**Published:** 2024-03-26

**Authors:** Tao He, Peijie Chen, Lu Jin, Jia Hu, Yifan Li, Liang Zhou, Shangqi Yang, Xiangming Mao, Yaoting Gui, Yun Chen, Yongqing Lai

Mol Med Rep 17: 2051–2060, 2018; DOI: 10.3892/mmr.2017.8052

Following the publication of the above article, the authors contacted the Editorial Office to explain that a couple of errors concerning data handling/labelling had been made, firstly during the preparation of the representative images in Fig. 3B, resulting in the wrong image being selected for the data panel showing the ACHN cells treated with ‘Inhibitor NC’ at 0 h experiment, and secondly in Fig. 5A, resulting in the wrong image being selected for the data panel showing the ACHN cells treated with ‘Inhibitor NC’ experiment. The authors requested that a corrigendum be published to take account of the errors that were made during the preparation of this figure.

Subsequently, an independent investigation of the published data was undertaken by the Editorial Office, which revealed that the ‘Inhibitor’ data panel in Fig. 6A and the ‘Mimic NC’ data panel in Fig. 6B were also overlapping, such that these data were likely to have been derived from the same original source, even though these data panels were intended to have shown the results from differently performed experiments. The Editor of *Molecular Medicine Reports* has considered the authors’ request to publish a corrigendum, but given the number of overlapping data panels that have been identified and the number of figures that would be in need of correction, the Editor has decided to decline the authors’ request to publish a corrigendum on account of an overall lack of confidence in the presented data, and instead has determined that the paper should be retracted. Upon receiving this news from the Editor, the authors accepted the Editor's decision. The Editor apologizes to the readership of the Journal for any inconvenience caused.

